# Leveraging the Global Influenza Surveillance and Response System for global respiratory syncytial virus surveillance—opportunities and challenges

**DOI:** 10.1111/irv.12672

**Published:** 2019-08-24

**Authors:** Shobha Broor, Harry Campbell, Siddhivinayak Hirve, Siri Hague, Sandra Jackson, Ann Moen, Harish Nair, Rakhee Palekar, Soatiana Rajatonirina, Peter G Smith, Marietjie Venter, Niteen Wairagkar, Maria Zambon, Thedi Ziegler, Wenqing Zhang

**Affiliations:** ^1^ Medicine and Health Sciences Shree Guru Gobind Singh Tricentenary University Gurugram India; ^2^ Usher Institute of Population Health Research and Informatics University of Edinburgh Edinburgh UK; ^3^ Global Influenza Program, Influenza Preparedness and Response World Health Organization Geneva Switzerland; ^4^ Department of Influenza Norwegian Institute of Public Health Oslo Norway; ^5^ Pan American Health Organization Washington DC USA; ^6^ African Region Office World Health Organization Brazzaville Republic of Congo; ^7^ MRC Tropical Epidemiology Group London School of Hygiene and Tropical Medicine London UK; ^8^ Center for Viral Zoonosis, Department of Medical Virology University of Pretoria Pretoria South Africa; ^9^ Bill and Melinda Gates Foundation Seattle WA USA; ^10^ Virus Reference Department Public Health England London UK; ^11^ Research Center for Child Psychiatry University of Turku Turku Finland

**Keywords:** respiratory syncytial virus, surveillance, strategy

## Abstract

**Background:**

Respiratory syncytial virus (RSV)‐associated acute lower respiratory infection is a common cause for hospitalization and hospital deaths in young children globally. There is urgent need to generate evidence to inform immunization policies when RSV vaccines become available. The WHO piloted a RSV surveillance strategy that leverages the existing capacities of the Global Influenza Surveillance and Response System (GISRS) to better understand RSV seasonality, high‐risk groups, validate case definitions, and develop laboratory and surveillance standards for RSV.

**Methods:**

The RSV sentinel surveillance strategy was piloted in 14 countries. Patients across all age groups presenting to sentinel hospitals and clinics were screened all year‐round using extended severe acute respiratory infection (SARI) and acute respiratory infection (ARI) case definitions for hospital and primary care settings, respectively. Respiratory specimens were tested for RSV at the National Influenza Centre (NIC) using standardized molecular diagnostics that had been validated by an External Quality Assurance program. The WHO FluMart data platform was adapted to receive case‐based RSV data and visualize interactive visualization outputs.

**Results:**

Laboratory standards for detecting RSV by RT‐PCR were developed. A review assessed the feasibility and the low incremental costs for RSV surveillance. Several challenges were addressed related to case definitions, sampling strategies, the need to focus surveillance on young children, and the data required for burden estimation.

**Conclusions:**

There was no evidence of any significant adverse impact on the functioning of GISRS which is primarily intended for virologic and epidemiological surveillance of influenza.

## 1 INTRODUCTION

Respiratory syncytial virus (RSV) is an important cause of respiratory infections, with the most severe infections occurring in young children, older adults, and adults with chronic co‐morbidities.^1^, ^2^ Acute lower respiratory infections (bronchiolitis and pneumonia) due to RSV are among the most common causes of hospital admission (estimated 3.2 million annually) and hospital deaths (estimated 59 600) in young children globally and are associated with a high hospitalization cost burden.^3^, ^4^ The overall number of deaths associated with RSV is less certain but has been estimated to be between 94 600 and 149 400 deaths in young children globally.^5^ RSV disease in young children has been associated with persisting sequelae such as recurrent wheeze and asthma later in childhood.^6^ The burden of severe disease and deaths from RSV in high‐risk adults remains uncertain but initial estimates suggest that influenza virus is more common than RSV, but RSV infection has a higher risk of death than seasonal influenza in adults.^7^ The RSV vaccine development landscape is evolving rapidly. There are currently several vaccine candidates and monoclonal antibodies in various stages of clinical trials.^8^, ^9^ The World Health Organization (WHO) Product Development for Vaccines Advisory Committee assessed RSV as the most promising new vaccine of potential major public health importance that may be ready for widespread implementation in the next 10 years.^10^ A gap analysis indicates that most data on RSV disease symptoms, treatment, and acute sequelae are from high‐income countries in contrast to the RSV burden distribution. RSV disease data from low‐ and middle‐income countries (LMICs) are improving, but additional data and granularity are needed particularly on disease in the first 6 months of life.^11^ The Gavi Vaccine Alliance approved RSV immunization products in its Vaccine Investment Strategy for 2021‐25 contingent on availability of a licenced product, a SAGE recommendation, WHO pre‐qualification, and meeting the financial assumptions used as the basis of the RSV investment case.^12^ Given the importance of RSV as a significant cause of hospitalization and death in young children, it is likthat a cost‐effective vaccine would be considered for inclusion in national immunization programs globally.

Most LMICs in Asia and Africa, where the RSV disease burden is likely to be the greatest, have little or no national data and low awareness of RSV as a cause of disease. Clinical guidelines for the management of lower respiratory infections in children in LMICs such as the WHO Integrated Management of Childhood Illness (IMCI) 13 do not advocate the use of RSV diagnostic testing as yet and routine hospital information systems do not generally report data on RSV which can inform national immunization program decision making. This gap in knowledge may impede evidence‐based policy decisions with respect to the potential introduction of any new RSV vaccine in LMIC.

The Global Influenza Surveillance and Response System (GISRS) coordinated by WHO and endorsed by national governments tests more than two million respiratory specimens annually to monitor the spread and evolution of influenza viruses through a network of about 150 well‐established laboratories in 114 countries representing 91% of the world's population. The GISRS network is ideally placed as a platform for the introduction of a more systematic testing for RSV associated with respiratory illness as it already uses molecular diagnostics on respiratory specimens and conditioned to report regular and disciplined reporting to a global platform. Countries in Latin America and the Caribbean region started testing for RSV by immunofluorescence. As RSV molecular diagnostics became widely available, many countries within the GISRS started testing for RSV and other respiratory viruses as a by‐product of influenza surveillance using the WHO‐recommended influenza‐like illness (ILI), acute respiratory infection (ARI), and severe acute respiratory infection (SARI) case definitions (Table 1). However, the use of case definitions primarily meant for influenza surveillance and a lack of standardized testing protocols for RSV were likely to provide biased results. A WHO consultative meeting of influenza and RSV experts in 2015 proposed to leverage GISRS as a cost‐effective, affordable, and sustainable platform for RSV surveillance to generate policy‐actionable data on RSV in advance of new RSV vaccines becoming available.^14^ The WHO Global Influenza Program developed a strategy for RSV surveillance based on GISRS that was rolled out as a 3‐year pilot project in 14 countries (Argentina, Australia, Brazil, Canada, Chile, Côte d'Ivoire, Egypt, India, Mongolia, Mozambique, Russian Federation, Thailand, South Africa, and United Kingdom of Great Britain and Northern Ireland) across the six WHO regions in 2017‐18.^15^ These countries were selected in consultation with the WHO regional offices based on existing and strong capacities in laboratory and epidemiological surveillance and willingness to participate in the pilot. The overall aim of the pilot was to investigate the suitability of the GISRS platform for RSV surveillance in a range of settings. This paper describes the strategy and discusses how various epidemiological and laboratory challenges were addressed.

**Table 1:** WHO‐recommended case definitions for RSV and influenza surveillance

	RSV	Influenza
Hospital‐based	Extended SARI onset within past 10 d cough (cough or shortness of breath) *In infants less than 6 mo age* Apnea (temporary cessation of breathing from any cause) Sepsis o fever more than 37.5°C or hypothermia o shock (lethargy, fast breathing, cold skin, prolonged capillary refill, or weak pulse), and o seriously ill without apparent cause	SARI onset within past 10 d cough (cough or shortness of breath) history of fever or measured fever of 38°C or more
Community‐based	ARI sudden onset At least one of cough sore throat shortness of breath coryza	ILI onset within past 10 d measured fever of 38°C or more, and cough

## 2 PILOTING THE STRATEGY FOR RSV SURVEILLANCE

The overall strategy aimed to leverage and build on the existing capacities of the well‐established GISRS to test for RSV without adversely affecting its vital role in influenza surveillance.^15^ The continuing commitment by governments to GISRS would facilitate the extension to ownership and sustainability of RSV in national disease surveillance in addition to influenza. The primary aim of establishing RSV surveillance was to better understand the epidemiological and virologic features of RSV circulation in a wide range of settings and generate evidence on seasonality, high‐risk groups, and disease burden to inform potential introduction of new RSV vaccines. The pilot project was also built to evaluate the performance of new case definitions for RSV surveillance, develop global laboratory, epidemiological and reporting standards for RSV surveillance, assess incremental surveillance and laboratory costs, and finally test the feasibility and evaluate any adverse impact on GISRS.

**Figure 1:**
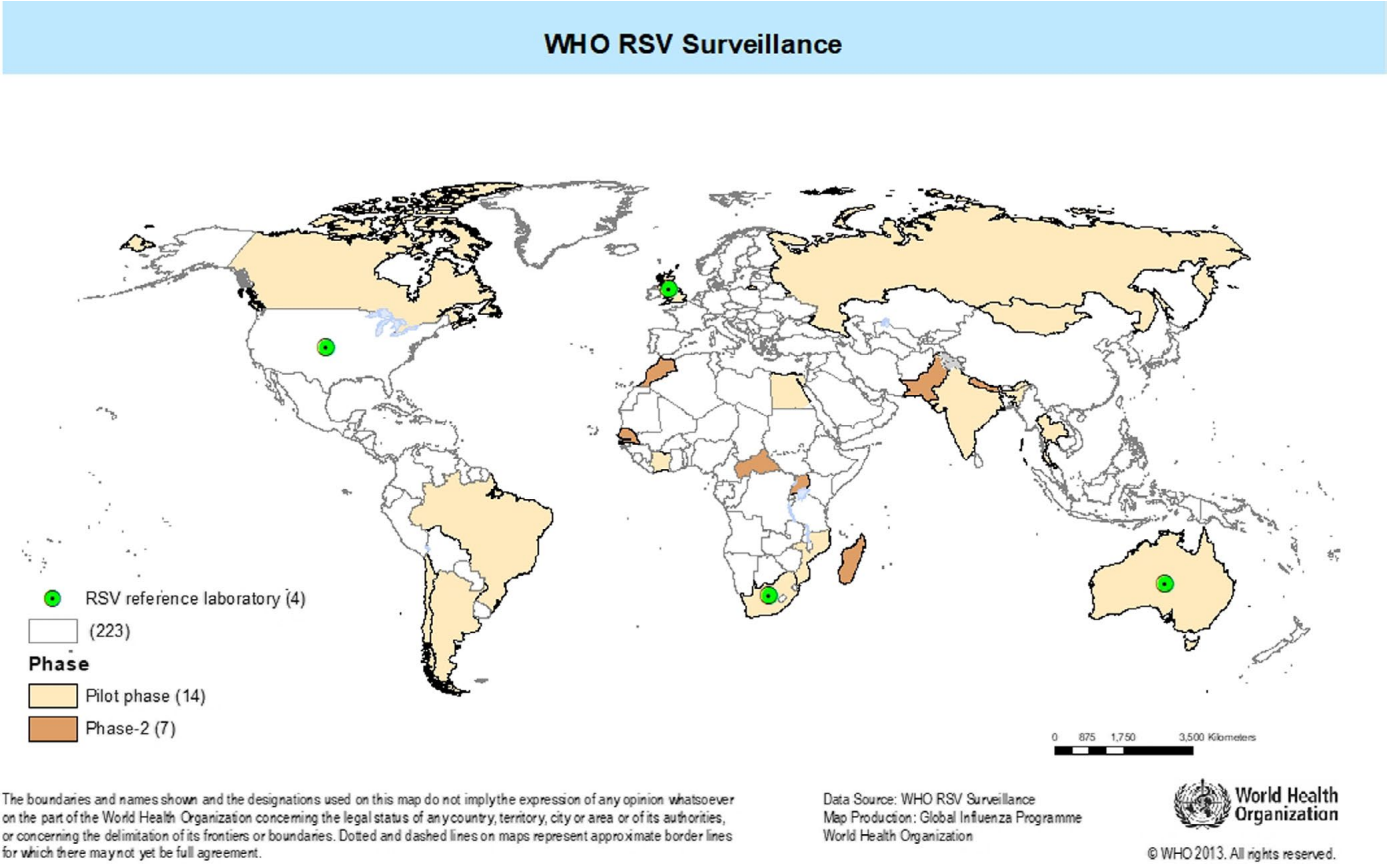
Countries participating in the WHO global RSV surveillance

**Figure 2:**
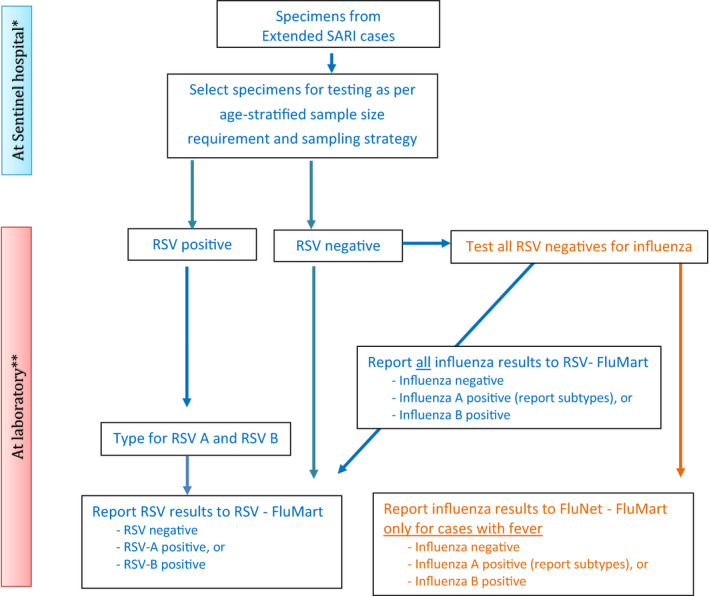
Algorithm for selection of patients for testing for RSV

Three designated reference laboratories (Respiratory Viruses Branch, Division of Viral Diseases, Centers for Disease Control and Prevention (CDC), United States; Public Health England, United Kingdom of Great Britain and Northern Ireland; and the National Institute of Communicable Diseases, South Africa) participated in the Quality Control for Molecular Diagnostics (QCMD) external quality assurance program for RSV. The National Influenza Centres (NICs) successfully participated in proficiency testing using panels developed by CDC containing both contemporary clades and past lineages of RSV. The NICs worked in conjunction with their disease control programs in fourteen countries (two or three from each of the six WHO regions) spread across both hemispheres (Figure 1), to screen patients in all age groups all year‐round, using extended SARI and ARI case definitions (Table 1) at sentinel hospitals and clinics, respectively (Figure 2). Respiratory specimens were collected and transported according to WHO guidelines 16 and batch‐tested at the NIC using a standardized real‐time RT‐PCR assay developed by CDC and validated against commonly used RNA extraction systems and amplification platforms. The NICs had the option of using either a commercial or an in‐house developed RT‐PCR assay provided it had been validated against the CDC assay. The use of enzymes and reagents for the RT‐PCR assay were standardized and supplied by the International Reagents Resource, CDC. The designated RSV reference laboratories provided technical and training support to the NICs on request. The WHO FluMart data reporting platform for influenza was adapted for countries to directly upload anonymized case‐based RSV data using their own database structure and system without having to transform it. Interactive data visualization of trends, distributions, and other outputs were developed that could be stratified by country, year, period, age, hospital or outpatient‐based surveillance, and presence or absence of fever. An external advisory group provided oversight to the pilot project.

## 3 OPPORTUNITIES AND CHALLENGES

The RSV surveillance strategy aims to leverage existing national resources of the GISRS without adversely impacting its established activities. The primary focus on hospital‐based surveillance, to ascertain more severe RSV infections, and on younger age groups, that are likely to be a key group driving vaccine policy decisions, placed some important practical demands on the pilot. The need to evaluate the performance of the extended SARI and ARI case definitions proposed for RSV surveillance required gathering case‐based clinical data in addition to routine surveillance data. However, it was not practical for detailed patient data to be collected in the pilot for a comprehensive clinical description of hospitalized RSV disease, nor could robust data be collected on population‐based RSV disease burden or disease costs.

### 3.1 Opportunities presented by the pilot

The pilot gave the opportunity to gather policy‐relevant data on RSV with a relatively low‐cost approach, building on the established GISRS platform. When effective RSV interventions become available, the data gathered, together with the estimates of economic and population burden of RSV, will enable Ministries of Health to make evidence‐based decisions on vaccine implementation and to monitor the impact of vaccination programs. This will have the added benefit that national support to GISRS is maintained and increased. Thus, the pilot represents an opportunity to influence emerging ideas on RSV surveillance and establish global guidelines and best practice.

Virologic surveillance in the future will allow for monitoring of any genetic drift and its relationship to antigenic change. These data could be used to identify escape mutants and assess the possible impact of genetic changes on virulence or vaccine or monoclonal efficacy. Work to develop capability and demonstration of the feasibility of generating high‐quality RSV sequence data could have important utility in responding to future calls for data after vaccine or monoclonal antibody introduction.

The RSV surveillance pilot has demonstrated a strong case for linking RSV and influenza surveillance systems with mutual benefits. Extension of surveillance to additional pediatric sites and age groups should result in important benefits to influenza surveillance as this group is currently underrepresented in GISRS data. Attention to data quality and completeness should also yield general benefits to GISRS. Combined RSV and influenza surveillance data also have the potential to increase the value of GISRS by generating data on RSV and influenza co‐infections and improving the certainty of vaccine effectiveness estimates for influenza, for which RSV disease presentation may be a clinical confounder. In the future, this platform could serve as a basis for a more broad‐based surveillance of respiratory pathogens.

Respiratory syncytial virus surveillance may not yield detailed and robust burden of disease estimates in many LMICs. Nevertheless, national policymakers should still find estimates of RSV‐associated hospitalizations useful in guiding policy in the absence of population‐based burden data. The pilot gave the opportunity to produce policy‐relevant RSV disease morbidity estimates and improve understanding of RSV seasonality and of global RSV virologic patterns—all of which will be of programmatic value in future national, regional, and global control programs.

Advocacy tools and approaches developed in the pilot, to raise awareness of RSV among the government, medical profession, and community, can be used in future communications, for example, to communicate the importance of influenza in different settings and age groups.

### 3.2 Epidemiology‐related challenges

Five major epidemiological concerns were identified for using GISRS for RSV surveillance. First, an ILI, ARI, or SARI case definition that requires fever would have low sensitivity to detect RSV, as over half of young children with RSV infection present without fever.^17^, ^18^ Furthermore, non‐respiratory presentations of RSV infection in very young infants are recognized (apnea, sepsis with no apparent cause) and a wider case definition would be needed in this age group.^19^, ^20^ The pilot addressed this concern by using extended SARI and ARI case definitions (which do not include fever) for hospital‐based and community‐based surveillance, respectively. In addition, the case definition for infants aged less than 6 months included apnea and sepsis from undetermined cause (Table 1). Second, the RSV disease burden is highest in children aged less than 2 years,^21^ an age group historically underrepresented in influenza surveillance by the GISRS. The pilot addressed this concern with quota sampling that required each country to recruit and test 1000 eligible patients per year (about 20 per week) stratified equally across four age groups viz. less than 6 months, 6 months to less than 5 years, 5 to less than 65 years, and 65 years and above. This would allow an RSV prevalence of 5%‐10% to be detected with a 95% confidence interval in each of the age groups. Countries were encouraged to add pediatric wards and hospitals, and neonatal and pediatric intensive care units to their existing GISRS sentinel sites. Third, RSV seasonality is not fully understood globally. Even where it is, seasonality may vary year to year and differ in timing from that of influenza 22, 23, 24; thus, GISRS sampling confined to the influenza season may miss many RSV cases. The pilot addressed this concern through year‐round surveillance for RSV in contrast to influenza surveillance which in some countries is restricted to the influenza season. Fourth, estimation of the population‐based burden from severe RSV disease requires knowledge of the denominator population from which cases are drawn. This was not available for most sites included in the pilot. However, countries were encouraged to gather data on all‐cause hospital admissions, and hospitalizations for respiratory disease, which provides an estimate, of the contribution of RSV to the hospital burden of disease. In addition, countries were requested, where possible, to estimate hospital catchment populations required for estimating population‐based burden.^15^, ^25^ Fifth, the pilot aimed to estimate the number of hospitalizations associated with RSV at each surveillance site. These data would indicate the hospital burden of RSV at that surveillance site and serve to estimate the proportional contribution of RSV‐associated hospitalization to all‐cause hospitalization. The total number of all‐cause and respiratory or SARI hospital admissions would generally be available. However, the number of extended SARI hospital admissions may not be routinely available to estimate the proportion of extended SARI patients included in the pilot. The assumption that recruited patients are the same as non‐recruited patients may not be true and hence may produce biased estimates of the RSV‐associated hospital burden. An alternate approach could be to count the number of respiratory admissions that needed to be screened over the same period to identify the number of extended SARI patients who were tested. This would give the proportion of all respiratory admissions that are identified as extended SARI patients and tested for RSV. This sampling fraction could then be used to adjust the number of extended SARI patients who were tested and were positive to give an estimate of the total number of RSV‐associated hospitalizations at the sentinel site. Ideally, the sampling fraction would be specified for each week, age group, and sentinel surveillance site. For surveillance sites where catchment population estimates are available, it may be possible to estimate population‐based denominators for severe RSV disease burden.^25^

### 3.3 Laboratory‐related challenges

Three major laboratory‐related concerns were considered. First, most NICs were already testing for RSV and other respiratory viruses using commercial or laboratory‐developed molecular diagnostics, either monoplex or multiplex RT‐PCR assays as well as antigen‐based testing methods that varied in sensitivity and specificity. Laboratories in Latin America were using direct or indirect immunofluorescence assay (IFA) to detect RSV, the sensitivity and specificity of which varies with the age of the patient, and type and quality of specimen. The sensitivity of IFA might be low for RSV detection especially in the off‐season period.^26^, ^27^ All laboratories agreed to use real‐time RT‐PCR for RSV detection. Although most switched to the CDC monoplex RT‐PCR assay, a few laboratories opted to continue using commercial or other laboratory‐developed RT‐PCR assays after validation against the CDC assay. Second, though important to collect information on the circulating RSV strain (A or B) to better understand their association with disease severity,^28^, ^29^, ^30^ the decision to type RSV was left to the individual laboratories, depending upon their available resources and expertise. Third, the decision to conduct genome sequencing of RSV was deferred to a later phase to prioritize establishing a surveillance system for RSV detection during the pilot phase, and to better define the goals of virologic surveillance of RSV.

## 4 DISCUSSION

The overarching aim of the pilot was to build on the successful global influenza surveillance platform, GISRS, to obtain RSV surveillance data without having a negative impact on GISRS. The primary motivation was to establish systems to obtain data to inform policy and decision making for possible future RSV vaccine investment, especially in LMIC settings in which it may be unlikely that there will be other relevant sources of suitable RSV data for this purpose. A midterm review assessed that it was feasible to leverage GISRS for RSV surveillance with little incremental cost without any significant adverse impact on the functioning of GISRS for influenza surveillance.

We have outlined the major challenges faced, and the decisions made to tackle these in the development of the RSV surveillance pilot approach. The pilot collected data to monitor performance at each site and will be able to report on the degree of success achieved using the approach adopted. For example, the pilot will report on the relative performance of the case definitions for the identification of RSV and influenza cases in hospital and primary care settings and will monitor the success in recruitment of cases in the specific targeted high‐risk groups. A limitation of the current pilot is that it did not specifically target pregnant women for RSV surveillance. Although two studies from Nepal and South Africa have reported that RSV infection during pregnancy does not adversely affect the pregnancy outcomes nor that post‐partum RSV infection of neonates cause severe disease,^31^ RSV surveillance in pregnant women should be considered for inclusion in some sites in future extensions of the global pilot. An important additional resource required (beyond that for GISRS) is the ability to report data from individual cases. This will help assess the validity of changes to the surveillance case definitions adopted and will help describe atypical case presentations, for example, in older adults and infants. This should help guide future decisions about how future RSV surveillance should be conducted and inform the design of studies of vaccine effectiveness. Initial feedback suggests that many sites will have to expand their existing GISRS hospital surveillance to include more pediatric hospitals, and those that admit neonates and young infants so that this important risk group is adequately represented. Enhanced data on respiratory support required during hospitalization will also assist in assessment of the health costs associated with RSV. These data will be important in health economical assessments of any intervention (such as vaccines). Equally, it will be important to raise awareness of the possibility of RSV infection in causing the respiratory disorders in older adults and those with chronic medical conditions.

Once RSV seasonality is better defined, it may be possible to limit site‐specific surveillance to high transmission seasons for both RSV and influenza. Once RSV case definitions have been evaluated, the need for ongoing individual case data will be reconsidered. Multiyear data from RSV surveillance will allow the study of evolutionary changes in RSV over time and in response to widespread vaccine introduction.

An important aspect of the pilot is the collection of surveillance data on RSV and influenza in both primary and secondary care settings. This is important as coinfection in causation of lower respiratory infections is increasingly recognized.^32^ This joint surveillance will help improve knowledge of RSV and influenza global transmission patterns and will inform future considerations of the need for and feasibility of broader infectious disease surveillance platforms which could encompass several other important respiratory pathogens that can result in hospitalization.

A major advantage of using the existing GISRS platform is that GISRS has built close relationships with Ministries of Health and policymakers in individual countries. This is clearly signaled by the financial support to the RSV surveillance pilot provided by national GISRS platforms. The pilot served to strengthen these relationships by providing policymakers with information on RSV from which vaccine investment, healthcare planning decision, and cost‐benefit analyses can be made in the future. We expect that these data raise awareness of RSV as one of the most common causes of hospitalization in young children globally and an underappreciated cause of disease in older adults. We will work closely with other stakeholders in each country to ensure that these data are presented to the appropriate audiences and respond to local comments and needs. Another possible approach to long‐term sustainability may be to integrate this surveillance within a broader (respiratory) infectious disease surveillance platform in secondary care that encompasses important infectious agents in a cost‐effective manner. The long‐term sustainability of RSV surveillance will depend on the success of relationships with the Expanded Program of Immunization and the Maternal, Neonatal and Child Health programs responsible for delivering RSV immunization products.

The pilot project should help define the minimum dataset and minimum surveillance effort required to maintain a functional and useful RSV surveillance, in a cost‐efficient manner and without adversely impacting on essential influenza surveillance. Information obtained should also help in better understanding the relative importance of RSV as a cause of healthcare burden in various risk groups and the overlap between influenza and RSV viral infection globally. It is hoped that experience from this pilot will serve as a basis from which to plan future RSV surveillance activities. Future extensions of the pilot could include incorporating additional high‐risk groups, such as pregnant women, and extending the global scale of RSV surveillance so that global transmission patterns and seasonality can be better studied.

Finally, the pilot phase did not track mortality or focus on severity of illness and did not follow up subjects for long‐term sequelae such as wheeze or asthma, or conduct surveillance in pregnant women to assess the impact of RSV infection on pregnancy and birth outcomes, which could be a limitation or an opportunity to explore, when RSV surveillance is established using lessons learnt from the pilot phase.

In conclusion, challenges notwithstanding, the pilot project demonstrated the feasibility of leveraging the GISRS for RSV surveillance without any significant negative impact on influenza surveillance.
